# Supplementary material for: Quantum control and Berry phase of electron spins in rotating levitated diamonds in high vacuum
Source: Nat Commun. 2024 Jun 13;15:5063. doi: 10.1038/s41467-024-49175-3 (PMC11176172; doi:10.1038/s41467-024-49175-3)
Supplement: Supplementary file 1 — supplementary information [file 41467_2024_49175_MOESM1_ESM.pdf]

Supplementary Information

Yuanbin Jin,[1] Kunhong Shen,[1] Peng Ju,[1] Xingyu Gao,[1]
Chong Zu,[2] Alejandro J. Grine,[3] and Tongcang Li[1,4,5,6,*]

[1] *Department of Physics and Astronomy, Purdue University, West Lafayette, Indiana 47907, USA*

[2] *Department of Physics, Washington University, St. Louis, MO, 63130, USA*

[3] *Sandia National Laboratories, P.O. Box 5800, Albuquerque, NM, 87185, USA*

[4] *Elmore Family School of Electrical and Computer Engineering, Purdue University, West Lafayette, Indiana 47907, USA*

[5] *Purdue Quantum Science and Engineering Institute, Purdue University, West Lafayette, Indiana 47907, USA*

[6] *Birck Nanotechnology Center, Purdue University, West Lafayette, Indiana 47907, USA*

(Dated: May 31, 2024)

SUPPLEMENTARY NOTE 1: EXPERIMENT SETUP AND SURFACE ION TRAP

Supplementary Figure 1 shows the schematic diagram of our experimental setup. The surface ion trap is fabricated on a sapphire wafer, which has a high transmittance for visible and near infrared laser. The chip is fixed on a 3D stage and installed in a vacuum chamber.

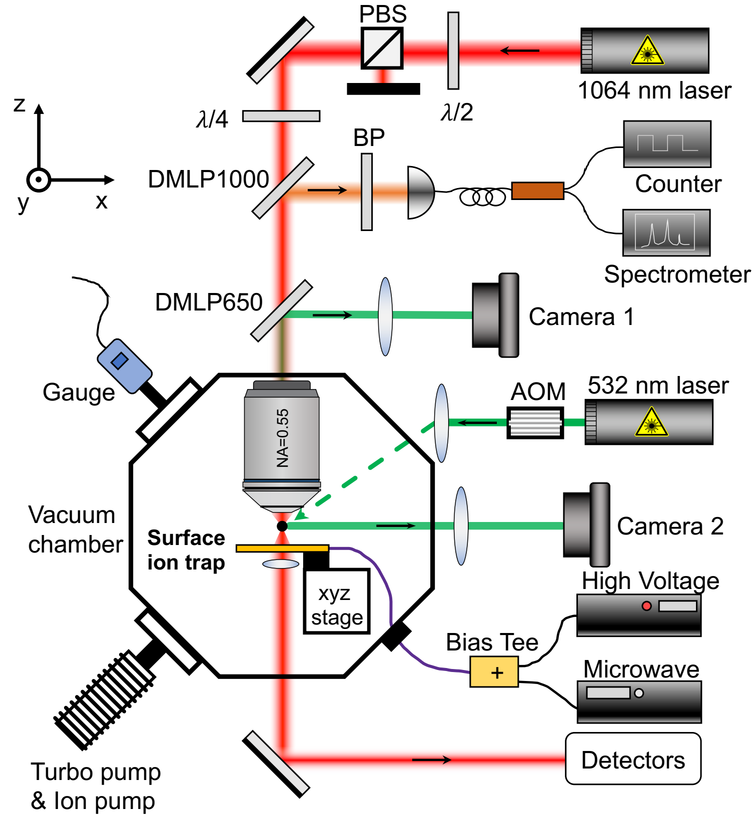

Supplementary Figure 1. Schematic diagram of the experimental setup. The surface ion trap is fixed on a 3D stage and installed in the vacuum chamber. A 532 nm laser beam controlled by an acousto-optic modulator (AOM) is incident from the bottom to excite diamond NV centers in a levitated nanodiamond. The photoluminescence (PL) is collected by an objective lens. A 1064 nm laser beam focused by the same objective lens is used to monitor the motion of levitated nanodiamonds. The PL is separated from the 532 nm laser and the 1064 nm laser by two long-pass dichroic mirrors (DMLP650 and DMLP1000). PBS: Polarizing beam splitter; $\lambda/2$: half-wave plate; $\lambda/4$: quarter-wave plate; BP: band-pass filters

* tcli@purdue.edu

The AC high voltage signal used to levitate nanodiamonds and the microwave used for quantum control are combined with a bias tee and delivered to the chip. A 532 nm laser beam is incident from the bottom to polarize a levitated nanodiamond. The photoluminescence (PL) is collected by an objective lens with a numerical aperture (NA) of 0.55. A 1064 nm laser beam focused by the same objective lens is used to monitor the center-of-mass (CoM) motion and the rotational motion of levitated nanodiamonds. The PL is separated from the 532 nm laser and the 1064 nm laser by two long-pass dichroic mirrors. The count rate and the optical spectrum of the PL are measured by a photon counter and a spectrometer. Two cameras monitor the procedure of particles loading and the trapping position of levitated nanoparticles.

Instead of a surface ring ion trap with concentric rings [1, 2], we use an Ω-shaped circuit to deliver both a high voltage for trapping and a microwave for controlling NV centers. A blank hole in the center allows the probing laser to travel through. The planar design conveniently provides six-directional detection. To theoretically calculate the trapping parameters, including the trapping position and the trapping depth, we approximate the surface ion trap (Supplementary Figure 2(a)) as a perfect ring ion trap (Supplementary Figure 2(b)). The area $a \leq r \leq b$ is connected to an AC high voltage driving signal with a frequency of f_d and an amplitude of V_d, while the remaining parts are grounded. For a surface ion trap, the motion of particles in z axis (perpendicular to the chip surface) is more critical. The motion equation of a nanoparticle along the z axis can be approximately written as [1]:

$$m\frac{d^2 z}{dt^2} = -QV_d \cos\left(2\pi f_d t\right) f\left(a, b\right)\left(z - z_0\right), \tag{1}$$

where m is the mass of the nanoparticle, Q is the charges, $z_0 = \sqrt{a^{4/3}b^{4/3}/\left(a^{2/3} + b^{2/3}\right)}$ is the trapping position located at the zero field point, and $f(a, b)$ is the geometric factor given by

$$f\left(a, b\right) = \sqrt{\frac{9(b^{2/3} - a^{2/3})^2(b^{2/3} + a^{2/3})^6}{a^{4/3}b^{4/3}(a^{4/3} + a^{2/3}b^{2/3} + b^{4/3})^5}}. \tag{2}$$

Eq. 1 shares the same forms as Mathieu equation. The trapping eigenfrequency along z direction can be solved as:

$$\omega_z = \frac{q}{2\sqrt{2}} 2\pi f_d = \frac{QV_d}{2\sqrt{2}\pi m f_d} f\left(a, b\right). \tag{3}$$

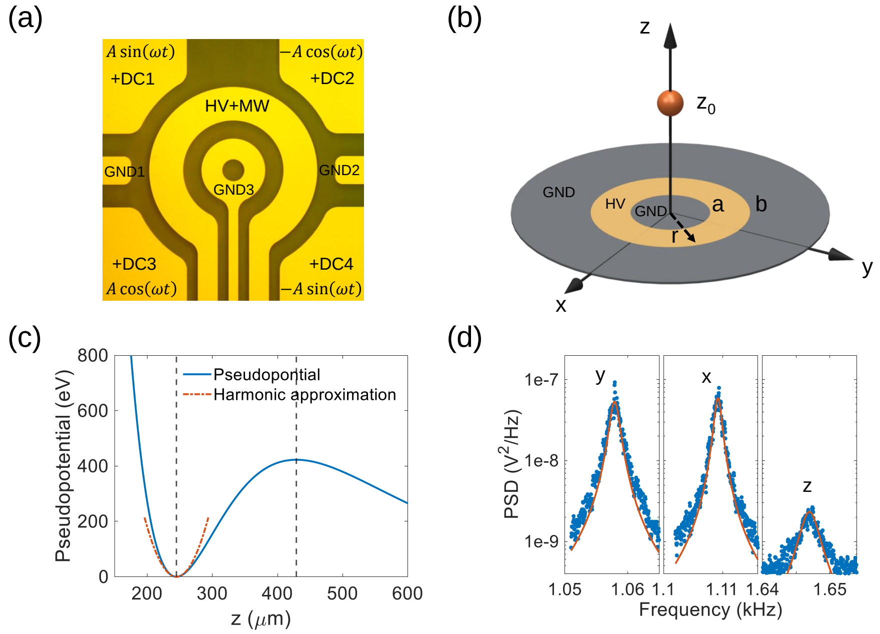

Supplementary Figure 2. Surface ion trap and CoM motion of a levitated nanodiamond. (a) Optical image of the surface ion trap. The outer four electrodes are used for compensation of residual charges on the chip surface. Trapping voltage and microwave can be delivered by a bias-tee simultaneously. (b) Equivalent surface ion trap with concentric rings design. Ring area $a \leq r \leq b$ is connected to an AC high voltage and the remaining parts are grounded. (c) Theoretical pseudopotential calculation in z axis of a concentric ring ion trap. The trapping position is located at $z_0 = 245$ μm and the trapping depth is around 420 eV. The parameters used for this calculation are listed in Supplementary Table I. (d) Power spectral densities (PSDs) of the CoM motion of the levitated nanodiamond in three dimensions at the pressure of 0.01 Torr. The radius is about 264 nm by the Lorentzian fitting.

For a regime with small displacement of a levitated nanodiamond, the electric potential can be approximated as a harmonic potential near the trapping region:

$$V_{p1}\left(z\right) = \frac{1}{2} m\omega_z^2 (z - z_0)^2 = \frac{Q^2 V_d^2}{16\pi^2 m f_d^2} f^2\left(a, b\right)\left(z - z_0\right)^2. \tag{4}$$

Generally, when the levitated nanodiamond moves away from the harmonic region, the pseudopotential can be written as [1]:

$$V_{p2}\left(z\right) = \frac{Q^2 V_d^2}{16\pi^2 m f_d^2} \left| \nabla \left(\frac{1}{\sqrt{1 + \left(a/z\right)^2}} - \frac{1}{\sqrt{1 + \left(b/z\right)^2}} \right) \right|^2. \tag{5}$$

Supplementary Table I. Parameters for the pseudopotential calculation of equivalent surface ion trap. q_z satisfies the condition of stable 3D trapping.

Q/e	R/nm	ρ/(kg/m^3)	V_d/V	f_d/Hz	a/μm	b/μm	q_z
2000	264	3500	300	$2\pi \times 1.6 \times 10^4$	270	450	0.29

The dimension of the surface ion trap is designed as $a = 270$ μm and $b = 450$ μm. We theoretically calculate the trapping potential of a levitated nanodiamond in z axis, as shown in Supplementary Figure 2(c). The red dash-dotted curve and blue solid curve are calculated by Eq. 4 and Eq. 5, respectively. All the parameters are summarized in Supplementary Table I. The theoretical trapping position z_0 is 245 μm, which is very close to the simulation result 253 μm for the current ion trap design. The difference is due to the asymmetric ion trap design.

The trapping potential is dependent on the eigenfrequency of a levitated particle, which is proportional to the charge to mass ratio (Q/m). Thus, it is necessary to increase the charge number carried on particles to achieve stable levitation in an ion trap. In our experiment, the diamond particles were purchased from Adamas Nano and the product model is MDNV1umHi10mg (1 micron Carboxylated Red Fluorescence, 1 mg/mL in DI Water, 3.5 ppm NV). These particles exhibit an average size of 750 nm. They are created by irradiating 2-3 MeV electrons on diamonds manufactured by static high-pressure, high-temperature (HPHT) synthesis and containing about 100 ppm of substitutional N. The nanodiamonds are first sprayed out by electrospray, which is supplied by a DC high voltage (~ 2 kV). Then the nanodiamonds are delivered to the trapping region of the surface ion trap with an extra linear Paul trap.

After a nanodiamond is trapped, we apply a 1064 nm laser to measure the CoM motion of the levitated nanodiamond. Supplementary Figure 2(d) is the power spectral densities (PSDs) of the CoM motion in x, y and z directions at the pressure of 0.01 Torr. The radius of the levitated nanodiamond is obtained to be about 264 nm based on the fitting of the PSDs. The experimental trapping frequency in the z direction is about $\omega_z/2\pi = 1.65$ kHz. Using Eq. 3, the charge number is estimated to be about 2000 for this nanodiamond. The surface ion trap creates an extremely deep potential well of 420 eV (Supplementary Figure 2(c)). According to our experimental results, the charge number of different levitated

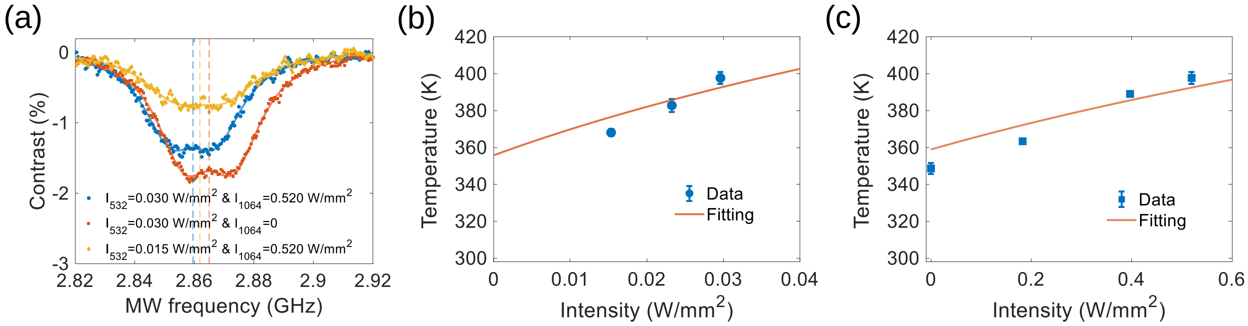

Supplementary Figure 3. Internal temperature of a levitated nanodiamond. (a) Optically detected magnetic resonances (ODMRs) of the levitated nanodiamond at different intensities of the 532 nm laser and the 1064 nm laser at the pressure of 1.3×10^{-5} Torr. (b) Internal temperature as a function of the intensity of the 532 nm laser. The intensity of the 1064 nm laser is 0.520 W/mm^2. (c) Internal temperature as a function of the intensity of the 1064 nm laser. The intensity of the 532 nm laser is 0.030 W/mm^2. Error bars in both (b) and (c) represent the standard deviation among three times measurements.

nanodiamonds varies from 1,000 to 10,000.

SUPPLEMENTARY NOTE 2: INTERNAL TEMPERATURE OF A LEVITATED NANODIAMOND

The heating induced by the absorption of the 532 nm laser and the 1064 nm laser affects the stability of the levitated nanodiamond in vacuum. Here we measure the optically detected magnetic resonances (ODMRs) of the levitated nanodiamond at different optical intensities of the 532 nm laser (I_{532}) and the 1064 nm laser (I_{1064}), as shown in Supplementary Figure 3(a). The pressure is fixed to 1.3×10^{-5} Torr. First, we adjust the intensity of the 532 nm laser from 0.015 W/mm^2 to 0.03 W/mm^2, while keeping $I_{1064} = 0.520$ W/mm^2. The internal temperature of the levitated nanodiamond is shown in Supplementary Figure 3(b). Then we measure the temperature when the intensity of the 1064 nm laser is changed from 0 to 0.520 W/mm^2 while the intensity of the 532 nm laser is fixed at $I_{532} = 0.03$ W/mm^2 (Supplementary Figure 3(c)). The red curves are the fittings by [3, 4]:

$$A_a = A_{gas} p \left(T - T_0\right) + A_{bb} \left(T^5 - T_0^5\right), \tag{6}$$

where the first term $A_a = \sum_\lambda \eta_\lambda I_\lambda V$ is the heating of the excitation laser ($\lambda = 532$ nm) and the probe laser ($\lambda = 1064$ nm), η_λ is the absorption coefficient of nanodiamond and I_λ is the laser intensity, V is the volume of nanodiamond. The second term is the cooling rate caused by gas molecule collisions, $A_{gas} = \frac{\kappa\pi R^2 v}{2T_0}\frac{\gamma'+1}{\gamma'-1}$, $\kappa \approx 1$ is the thermal accommodation coefficient of diamond, $R = 332$ nm is the radius of the nanodiamond used in this temperature measurement experiment, v is the mean thermal speed of gas molecules, γ' is the specific heat ratio ($\gamma' = 7/5$ for air near room temperature), p is the pressure, T_0 is the thermal temperature (298 K). Plug in the parameters, we get the coefficient A_{gas} to be 1.74×10^{-12} $m^3 \cdot s^{-1} \cdot K^{-1}$ for this nanodiamond. The last term is for cooling due to black-body radiation, where $A_{bb} = 72\zeta(5) V k_B^5 / \left(\pi^2 c^3 \hbar^4\right) \mathrm{Im}\left(\frac{\varepsilon-1}{\varepsilon+2}\right)$, $\zeta(5) \approx 1.04$ is the Riemann zeta function, k_B is the Boltzmann constant, c is the vacuum light speed, $\hbar$ is the reduced Planck's constant, ε is a constant and time-independent permittivity of nanodiamond across the black-body radiation spectrum. Based on the coefficient A_{gas}, we can calculate the absorption coefficients of 532 nm laser and 1064 nm laser to be 111 cm^{-1} and 5.87 cm^{-1}, respectively.

SUPPLEMENTARY NOTE 3: ROTATION OF A LEVITATED NANODIAMOND DRIVEN BY A ROTATING ELECTRIC FIELD

In our experiment, we use a rotating electric field to drive a levitated nanodiamond to rotate at a high speed. The four electrodes at the corners are applied with four sinusoidal signals with the same frequency and amplitude but $\pi/2$ phase difference to generate a rotating electric field. The two grounded electrodes labeled by GND1 and GND2 (Supplementary Figure 2(a)) are introduced to cancel the z component of the rotating electric field and make the electric field more symmetric. We simulate the electric fields for both the trapping potential and the rotating field using the COMSOL software. The simulation of the electric potential in xy-plane at different rotation phases are shown in Supplementary Figure 4. The rotation phases are changed from 0 to 2π by steps of $\pi/4$ in the simulation. The dipole moment ($\mathbf{p}$) of a levitated nanodiamond is aligned to the direction of the electric field ($\mathbf{E}_{xy}$) by the torque

$$\mathbf{M}_{electric} = \mathbf{p} \times \mathbf{E}_{xy} = |\mathbf{p}|\,|\mathbf{E}_{xy}| \sin\beta \cdot \mathbf{z}, \tag{7}$$

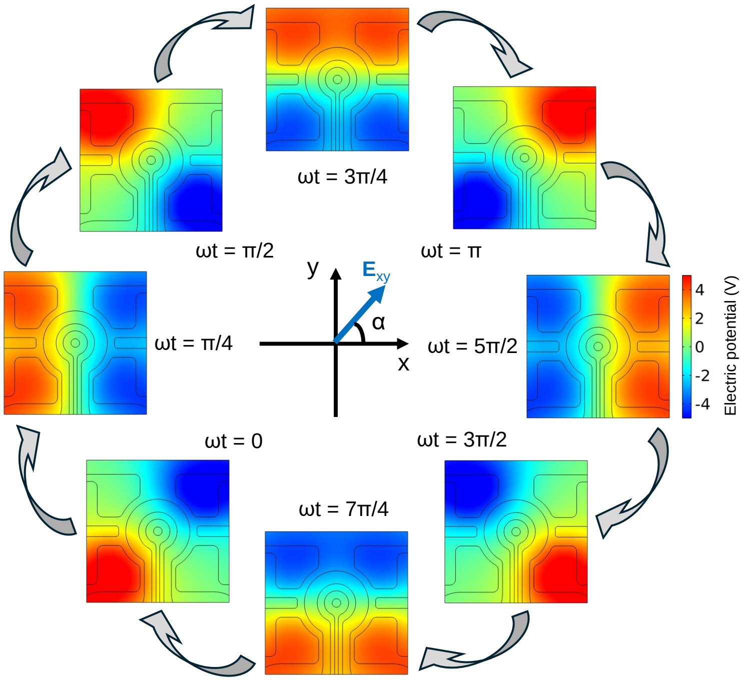

Supplementary Figure 4. Simulation of the rotating electric potential in xy-plane generated by the four electrodes. The rotation phase (ωt) of each figure is changed from 0 to 2π by the step of $\pi/4$, and the direction of corresponding electric field rotate following the rotation phase.

where β is the angle between the dipole moment and the electric field, **z** is the unit vector along z direction.

Based on the simulation as shown in Supplementary Figure 4, the amplitude and the direction (α) of the electric field in the xy-plane can be calculated, which is displayed in Supplementary Figure 5(a) and 5(b). $\alpha = 0$ indicates that the rotating electric field points to the positive x direction. The blue circles and red squares are the simulations with and without the compensation electrodes GND 1 and GND 2. Ideally, the direction of the rotating electric field should be $\alpha\,(t) = \pi/4 - \omega t$ (orange dashed curve). The inset of Supplementary Figure 5(b) is the asynchrony between the simulation result and an ideal rotation field with and without the compensation electrodes. The orientation of the electric field does not perfectly rotate at a constant speed in one period without the compensation electrodes (red squares). The maximum deviation is 5.5°. It hurts the stability of the rotational motion of the levitated nanodiamonds and expands the linewidth of nanodiamond's rotation signal. Moreover, the E_z component of the rotating electrical field oscillates with a large amplitude if no compensation electrode exits (Supplementary Figure 5(c)). The E_z component drives

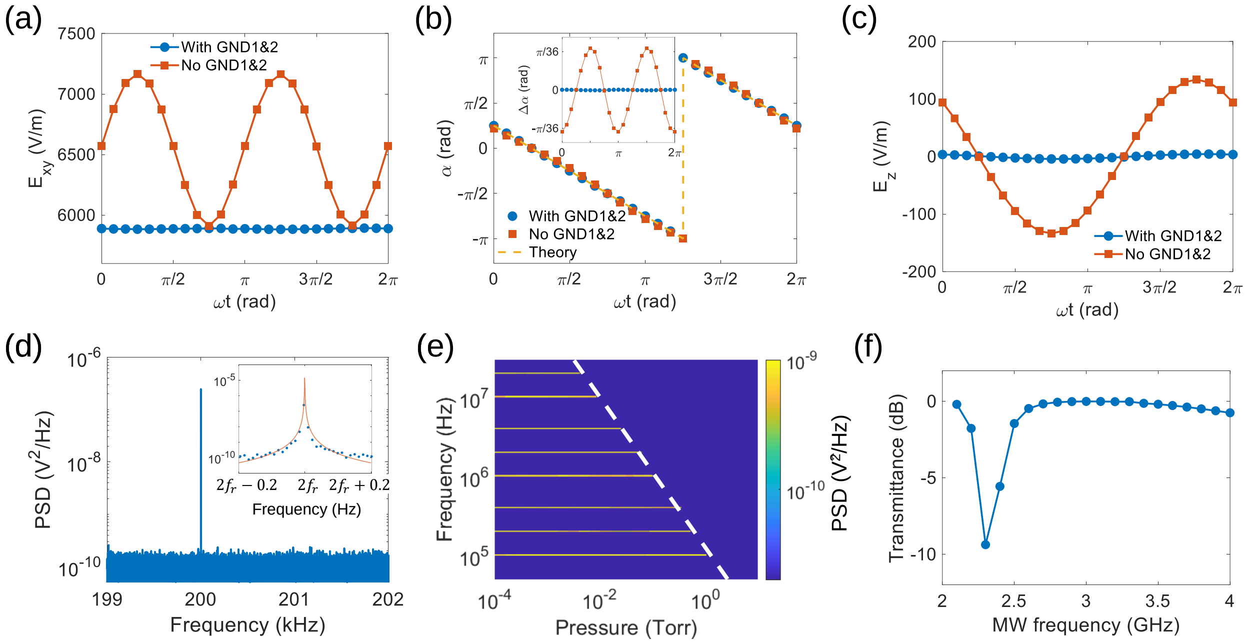

Supplementary Figure 5. Simulation of the rotating electric field and the rotational motion of a levitated nanodiamond. Time evolution of the xy-plane component (a) and direction (b), z component (c) of the electric field with (blue circles) and without (red squares) the compensation electrodes of GND1 and GND2. α describes the orientation of the electric field ($\alpha = 0$ indicates the electric field points to positive x direction). (d) PSD of the rotational motion at the rotation frequency of 0.1 MHz. The linewidth is 9.9×10^{-5} Hz by the fitting (inset). The ratio of the center frequency to the linewidth is 2×10^9. (e) PSDs of the rotational motion of the levitated nanodiamond at different pressures, showing the maximum rotation frequency at different pressures. The rotation frequencies are 0.05 MHz, 0.1 MHz, 0.2 MHz, 0.5 MHz, 1 MHz, 2 MHz, 5 MHz and 10 MHz, respectively. The upper limit of the rotation frequency is inversely proportional to pressure with the electric field driving. Particles stop rotating above the dashed white line. (f) Simulation of microwave transmittance of the surface ion trap.

a levitated nanodiamond to oscillate in the z direction, causing the loss of the levitated nanodiamond in high vacuum. The two compensation electrodes effectively solve these issues. The transmittance of a microwave through the Ω-shaped circuit is simulated (Supplementary Figure 5(f)) to ensure the microwave has low loss for frequencies from 2.6 GHz to 3.1 GHz.

Then we drive a levitated nanodiamond to rotate using the rotating electric field. The

PSD of the rotational motion at the rotation frequency of 0.1 MHz is shown in Supplementary Figure 5(d). The linewidth of the rotation signal is about 9.9×10^{-5} Hz based on a Lorentzian fitting (inset of Supplementary Figure 5(d)). Thus, the ratio of the center frequency to the linewidth is 2×10^{9}, demonstrating the rotational motion is ultra-stable with easy control by this method.

Meanwhile, the rotational motion of the levitated nanodiamond is damped by the interaction with the remaining gas molecules in vacuum chamber. The damping torque of a sphere is [5, 6]

$$M_{gas} = -I\omega_r\gamma_d, \tag{8}$$

where I is the moment of inertia of the nanodiamond, ω_r is the angular velocity, $\gamma_d = 40\eta' pR^2/3mv$ is the damping rate of rotational motion, $\eta' \approx 1$ is the accommodation factor accounting for the efficiency of the angular momentum transferred onto the nanodiamond by gas molecule collisions, p is the pressure, R and m are the radius and the mass of the particle, v is the mean thermal speed of gas molecules. Thus, the rotational motion equation can be written as:

$$I\frac{d\omega_r}{dt} = M_{electric} + M_{gas}. \tag{9}$$

The maximum rotation frequency of the levitated nanodiamonds is obtained at $M_{electric} = -M_{gas}$ and $\beta = \pi/2$, which is limited by the pressure in the vacuum chamber. The maximum rotation frequency at a certain pressure is:

$$\omega_{r,\max} = |\mathbf{p}|\,|\mathbf{E}_{xy}|\,/I\gamma_d. \tag{10}$$

We measure the upper limit of the rotation frequency at different pressures (Supplementary Figure 5(e)). The PSDs as functions of air pressure are measured at the rotation frequencies of 0.05 MHz, 0.1 MHz, 0.2 MHz, 0.5 MHz, 1 MHz, 2 MHz, 5 MHz and 10 MHz. The levitated nanodiamond stops rotating when the pressure is too large for that rotation frequency. The maximum rotation frequency is inversely proportional to the pressure (white dashed curve). The dipole moment of the nanodiamond ($R = 264$ nm) is estimated to be $|\mathbf{p}| = 3.13\times10^{-25}$ C·m (1.96 e·μm). We can adjust and lock the rotation of the levitated nanodiamond at arbitrary frequency and pressure in the region below the white dashed curve. The maximum rotation frequency is $\omega_r = 2\pi\times20$ MHz in this experiment, which is limited

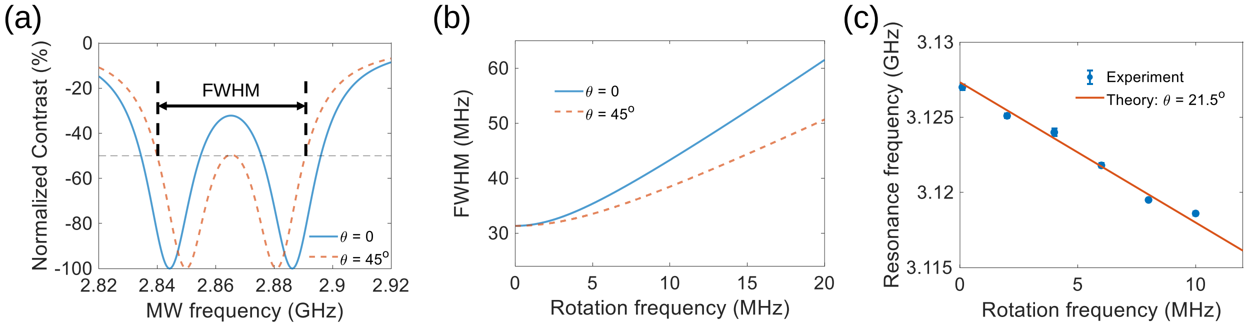

Supplementary Figure 6. Berry phase induced by a rotating nanodiamond. (a) Theoretically calculated ODMR of NV centers with different orientations at the rotation frequency of 20 MHz. The blue solid and red dashed lines are calculated at $\theta = 0$ and $\theta = 45°$, respectively. (b) Theoretically calculated FWHM of the ODMR as a function of rotation frequency. The blue solid curve and the red dashed curve are calculated for $\theta = 0$ and $\theta = 45°$, respectively. (c) Experimental results of the frequency shift due to the Berry phase induced by counterclockwise rotation (blue circles), and theoretical calculated resonance frequency as a function of the rotation frequency at $\theta = 21.5°$ (red curve). Error bars show the standard deviation among three measurements.

by the π-phase shifter (Mini-Circuits, ZFSCJ-2-2-S) used to generate the signals on the four electrodes. Under favorable conditions, the rotational motion can achieve a frequency exceeding 10 GHz at the pressure of 10^{-6} Torr based on the dashed line in Supplementary Figure 5(e).

SUPPLEMENTARY NOTE 4: BERRY PHASE OF ROTATING NV ELECTRON SPINS

A. Without an external magnetic field

In a rotating diamond, the embedded NV centers follow the rotation of the particle with an angular frequency of ω_r. Considering an arbitrary NV center in a diamond at the time of t, the angle between the NV axis and z axis is θ, and the azimuth angle is $\phi(t) = \omega_r t$ relative to x axis. In the absence of an external magnetic field and neglecting strain effects,

the Hamiltonian of the rotating NV center in the laboratory frame can be written as [7]

$$\begin{aligned} H_{0,lab} &= \tfrac{1}{\hbar} R\left(t\right) D S_z^2 R^{\dagger}\left(t\right) = \tfrac{1}{\hbar} e^{-i\phi S_z} e^{-i\theta S_y} D S_z^2 e^{i\theta S_y} e^{i\phi S_z} \\ &= D\hbar \begin{pmatrix} \cos^2\theta + \frac{\sin^2\theta}{2} & \frac{e^{-i\phi}\cos\theta\sin\theta}{\sqrt{2}} & \frac{e^{-2i\phi}\sin^2\theta}{2} \\ \frac{e^{i\phi}\cos\theta\sin\theta}{\sqrt{2}} & \sin^2\theta & -\frac{e^{-i\phi}\cos\theta\sin\theta}{\sqrt{2}} \\ \frac{e^{2i\phi}\sin^2\theta}{2} & -\frac{e^{i\phi}\cos\theta\sin\theta}{\sqrt{2}} & \cos^2\theta + \frac{\sin^2\theta}{2} \end{pmatrix} \end{aligned}, \tag{11}$$

where D is the zero-field splitting, $R\left(t\right) = R_z\left(\phi\left(t\right)\right) R_y\left(\theta\right)$ is the rotation transformation, and $R_y\left(\theta\right) = \exp\left(-i\theta S_y\right)$ $\left(R_z\left(\phi\right) = \exp\left(-i\phi S_z\right)\right)$ expresses the rotation of spin around the y (z) axis in terms of θ (ϕ), $\mathbf{S}$ is the spin operator. The Hamiltonian possesses three eigenstates $|m_s, t\rangle_{lab} = R\left(t\right)|m_s, 0\rangle_{lab}$ ($m_s = 0, \pm 1$),

$$\begin{aligned} |1, t\rangle_{lab} &= R\left(t\right)\begin{pmatrix} 1 \\ 0 \\ 0 \end{pmatrix} = \begin{pmatrix} e^{-i\phi}\cos^2\frac{\theta}{2} \\ \frac{\sin\theta}{\sqrt{2}} \\ e^{i\phi}\sin^2\frac{\theta}{2} \end{pmatrix} \\ |0, t\rangle_{lab} &= R\left(t\right)\begin{pmatrix} 0 \\ 1 \\ 0 \end{pmatrix} = \begin{pmatrix} -\frac{e^{-i\phi}\sin\theta}{\sqrt{2}} \\ \cos\theta \\ \frac{e^{i\phi}\sin\theta}{\sqrt{2}} \end{pmatrix} \\ |-1, t\rangle_{lab} &= R\left(t\right)\begin{pmatrix} 0 \\ 0 \\ 1 \end{pmatrix} = \begin{pmatrix} e^{-i\phi}\sin^2\frac{\theta}{2} \\ -\frac{\sin\theta}{\sqrt{2}} \\ e^{i\phi}\cos^2\frac{\theta}{2} \end{pmatrix} \end{aligned}. \tag{12}$$

For a quantum system in an eigenstate, the system remains in the eigenstate and acquires a phase factor during an adiabatic evolution of the Hamiltonian. This factor arises from both the state's time evolution and the variation of the eigenstate with the changing Hamiltonian. The second term specifically corresponds to the Berry phase. Hence, the expression for the time-dependent spin state is [8]

$$e^{i\gamma_{m_s}} e^{-iH_{0,lab}t/\hbar}|m_s, t\rangle_{lab} = e^{i\gamma_{m_s}} e^{-iH_{0,lab}t/\hbar} e^{-i\phi S_z} e^{-i\theta S_y}|m_s, 0\rangle_{lab}, \tag{13}$$

where γ_{m_s} is the Berry phase. Here, the diamond particle rotates around the z axis with a constant θ, the Berry phase can be calculated as [8]

$$\gamma_{m_s} = i\int_0^t {}_{lab}\langle m_s, t'|\frac{\partial}{\partial t'}|m_s, t'\rangle_{lab} dt' = m_s \omega_r t\cos\theta. \tag{14}$$

The Berry phase of Eq. 14 is calculated for an open-path, which is gauge-dependent. However, for a closed loop, the Berry phase is gauge-invariant and can be expressed as $m_s\left[-2\pi\left(1-\cos\theta\right)\right]$. The result is equivalent to Eq. 14 of $m_s\left(2\pi\cos\theta\right)$.

The spin state of the NV center is observed through the interaction with a microwave magnetic field. In our experiment, the direction of the microwave is in the yz-plane and forms a slight angle θ' relative to the z axis, resulting from the asymmetric design of the waveguide. The Hamiltonian of the microwave in the laboratory frame can be written as

$$H_{MW,lab} = g\mu_B B_{MW}\cos\left(\omega_{MW}t\right)\left(S_z\cos\theta' + S_y\sin\theta'\right) = H_{MW,z,lab} + H_{MW,y,lab}, \tag{15}$$

which contains two components, the longitudinal term $H_{MW,z,lab} = g\mu_B B_{MW}\cos\left(\omega_{MW}t\right)S_z\cos\theta'$ and the transverse term $H_{MW,y,lab} = g\mu_B B_{MW}\cos\left(\omega_{MW}t\right)S_y\sin\theta'$. First, we consider the longitudinal term $H_{MW,z,lab}$. The expected value of the spin states can be expressed as

$$\begin{aligned}
&{}_{lab}\left\langle \pm 1, t\right| e^{iH_{0,lab}t/\hbar}e^{-i\gamma_{\pm 1}}H_{MW,z,lab}e^{i\gamma_0}e^{-iH_{0,lab}t/\hbar}\left|0,t\right\rangle_{lab} \\
&= g\mu_B B_{MW}\cos\left(\omega_{MW}t\right)\cos\theta'{}_{lab}\left\langle \pm 1, 0\right| e^{i\theta S_y}e^{i\phi S_z}e^{iH_{0,lab}t/\hbar}e^{-i\gamma_{\pm 1}}S_z e^{i\gamma_0}e^{-iH_{0,lab}t/\hbar}e^{-i\phi S_z}e^{-i\theta S_y}\left|0,0\right\rangle_{lab} \\
&= g\mu_B B_{MW}\cos\left(\omega_{MW}t\right)\cos\theta' e^{-i(\gamma_{\pm 1}-\gamma_0)}e^{i(E_{\pm 1}-E_0)t/\hbar}{}_{lab}\left\langle \pm 1, 0\right| e^{i\theta S_y}e^{i\phi S_z}S_z e^{-i\phi S_z}e^{-i\theta S_y}\left|0,0\right\rangle_{lab} \\
&= \tfrac{1}{2}g\mu_B B_{MW}\cos\theta'\left(e^{i\omega_{MW}t}+e^{-i\omega_{MW}t}\right)e^{\mp i\omega_r t\cos\theta}e^{iDt}{}_{lab}\left\langle \pm 1, 0\right| e^{i\theta S_y}S_z e^{-i\theta S_y}\left|0,0\right\rangle_{lab} \\
&= \tfrac{1}{2}g\mu_B B_{MW}\cos\theta'\left[e^{i(\omega_{MW}\mp\omega_r\cos\theta+D)t}+e^{i(-\omega_{MW}\mp\omega_r\cos\theta+D)t}\right]_{lab}\left\langle \pm 1, 0\right| e^{i\theta S_y}S_z e^{-i\theta S_y}\left|0,0\right\rangle_{lab} \\
&\approx \tfrac{1}{2}g\mu_B B_{MW}\cos\theta' e^{i(-\omega_{MW}+D\mp\omega_r\cos\theta)t}{}_{lab}\left\langle \pm 1, 0\right| e^{i\theta S_y}S_z e^{-i\theta S_y}\left|0,0\right\rangle_{lab}
\end{aligned}, \tag{16}$$

where the E_{m_s} is the corresponding eigenvalue of the Hamiltonian $H_{0,lab}$ for the spin state $|m_s, t\rangle$. According to Eq. 16, the transformation of spin states from $|m_s = 0\rangle_{lab}$ to $|m_s = \pm 1\rangle_{lab}$ can be driven by a microwave operating at the resonance frequency of $D \mp \omega_r\cos\theta$, where the frequency shift $\mp\omega_r\cos\theta$ is attributed to the Berry phase.

Regarding the second part of the Hamiltonian in the transverse direction, $H_{MW,y,lab}$, the interaction between the microwave and the spin states can be formulated as

$$\begin{aligned}
&{}_{lab}\left\langle \pm 1, t\right| e^{iH_{0,lab}t/\hbar}e^{-i\gamma_{\pm 1}}H_{MW,y,lab}e^{i\gamma_0}e^{-iH_{0,lab}t/\hbar}\left|0,t\right\rangle_{lab} \\
&= g\mu_B B_{MW}\cos\left(\omega_{MW}t\right)\sin\theta'{}_{lab}\left\langle \pm 1, 0\right| e^{i\theta S_y}e^{i\phi S_z}e^{iH_{0,lab}t/\hbar}e^{-i\gamma_{\pm 1}}S_y e^{i\gamma_0}e^{-iH_{0,lab}t/\hbar}e^{-i\phi S_z}e^{-i\theta S_y}\left|0,0\right\rangle_{lab} \\
&= g\mu_B B_{MW}\cos\left(\omega_{MW}t\right)\sin\theta' e^{-i(\gamma_{\pm 1}-\gamma_0)}e^{i(E_{\pm 1}-E_0)t/\hbar}{}_{lab}\left\langle \pm 1, 0\right| e^{i\theta S_y}e^{i\phi S_z}S_y e^{-i\phi S_z}e^{-i\theta S_y}\left|0,0\right\rangle_{lab} \\
&= \tfrac{1}{2}g\mu_B B_{MW}\sin\theta'\left(e^{i\omega_{MW}t}+e^{-i\omega_{MW}t}\right)e^{\mp i\omega_r t\cos\theta}e^{iDt} \\
&\times{}_{lab}\left\langle \pm 1, 0\right| e^{i\theta S_y}\tfrac{1}{2i}\left(S_+e^{i\omega_r t}-S_-e^{-i\omega_r t}\right)e^{-i\theta S_y}\left|0,0\right\rangle_{lab}
\end{aligned}. \tag{17}$$

The expected values are written as

$$\begin{aligned}
&{}_{lab}\left\langle +1,t\right| e^{iH_{0,lab}t/\hbar}e^{-i\gamma_{+1}}H_{MW,y,lab}e^{i\gamma_0}e^{-iH_{0,lab}t/\hbar}\left|0,t\right\rangle_{lab}\\
&=\tfrac{1}{4i}g\mu_B B_{MW}\sin\theta'\left[e^{i(\omega_{MW}-\omega_r\cos\theta+D+\omega_r)t}+e^{i(-\omega_{MW}-\omega_r\cos\theta+D+\omega_r)t}\right]\\
&\times{}_{lab}\left\langle +1,0\right| e^{i\theta S_y}S_+e^{-i\theta S_y}\left|0,0\right\rangle_{lab}\\
&\approx\tfrac{1}{4i}g\mu_B B_{MW}\sin\theta' e^{i(-\omega_{MW}+D+\omega_r-\omega_r\cos\theta)t}{}_{lab}\left\langle +1,0\right| e^{i\theta S_y}S_+e^{-i\theta S_y}\left|0,0\right\rangle_{lab}
\end{aligned},\qquad(18)$$

$$\begin{aligned}
&{}_{lab}\left\langle -1,t\right| e^{iH_{0,lab}t/\hbar}e^{-i\gamma_{-1}}H_{MW,y,lab}e^{i\gamma_0}e^{-iH_{0,lab}t/\hbar}\left|0,t\right\rangle_{lab}\\
&=-\tfrac{1}{4i}g\mu_B B_{MW}\sin\theta'\left[e^{i(\omega_{MW}+\omega_r\cos\theta+D-\omega_r)t}+e^{i(-\omega_{MW}+\omega_r\cos\theta+D-\omega_r)t}\right]\\
&\times{}_{lab}\left\langle -1,0\right| e^{i\theta S_y}S_-e^{-i\theta S_y}\left|0,0\right\rangle_{lab}\\
&\approx-\tfrac{1}{4i}g\mu_B B_{MW}\sin\theta' e^{i(-\omega_{MW}+D-\omega_r+\omega_r\cos\theta)t}{}_{lab}\left\langle -1,0\right| e^{i\theta S_y}S_-e^{-i\theta S_y}\left|0,0\right\rangle_{lab}
\end{aligned}.\qquad(19)$$

Utilizing Eq. 18 and Eq. 19, the resonance frequency of microwave for transforming the spin state from $|m_s=0\rangle_{lab}$ to $|m_s=\pm1\rangle_{lab}$ is $D\pm\omega_r(1-\cos\theta)$. In addition to the frequency shift of $\mp\omega_r\cos\theta$ caused by the Berry phase, there is another term of ω_r coming from the rotational Doppler effect [8]. In our experiment, the angle θ' of the microwave relate to the z axis is approximately 8.5°. Consequently, the dominant transition probability arises from the longitudinal component, characterized by a frequency shift of $\mp\omega_r\cos\theta$ due to the Berry phase.

The energy levels of NV centers with four orientations are degenerate in the absence of an external magnetic field. When the nanodiamond undergoes rotation, the electron spin resonance frequency experiences a shift due to the Berry phase, and this shift depends on the angle θ between the NV axis and the rotation axis. The electron spin resonance frequencies of NV spins along different orientations become non-degenerate. The ODMR of NV at different orientations are theoretically calculated by Eq. 16 at the rotation frequency of 20 MHz (Supplementary Figure 6(a)). The orientations corresponding to the blue solid curve and the red dashed curve are $\theta=0°$ and $\theta=45°$, respectively. The intrinsic linewidth is $2\pi\times19$ MHz, and the strain effect splitting E is $2\pi\times6.7$ MHz. The eight dips are not separated in the ODMR spectrum at a rotation frequency of a few MHz because of the large linewidth. Here we use the FWHM parameter of the ODMR spectrum to indicate the frequency shift by the Berry phase of a rotating NV center. The FWHM of the ODMR is mainly determined by the splitting of the NV centers that have the smallest θ. Supplementary Figure 6(b) shows the FWHM of the ODMR as a function of rotation frequency. The NV centers, which have the smallest θ, show the highest sensitivity of the frequency shift due to the Berry phase.

B. With an external magnetic field

To precisely measure the frequency shift induced by the Berry phase of a rotating NV center, an external magnetic field **B** along the z direction can be applied. This serves to distinguish the energy levels of NV centers in four different orientations. The Hamiltonian of a NV center in the laboratory frame with the external magnetic field can be expressed as

$$\begin{aligned} H_{lab} &= H_{0,lab} + g\mu_B B S_z = \frac{1}{\hbar} e^{-i\phi S_z} e^{-i\theta S_y} D S_z^2 e^{i\theta S_y} e^{i\phi S_z} + g\mu_B B S_z \\ &= D\hbar \begin{pmatrix} \cos^2\theta + \frac{\sin^2\theta}{2} + \frac{g\mu_B B}{D} & \frac{e^{-i\phi}\cos\theta\sin\theta}{\sqrt{2}} & \frac{e^{-2i\phi}\sin^2\theta}{2} \\ \frac{e^{i\phi}\cos\theta\sin\theta}{\sqrt{2}} & \sin^2\theta & -\frac{e^{-i\phi}\cos\theta\sin\theta}{\sqrt{2}} \\ \frac{e^{2i\phi}\sin^2\theta}{2} & -\frac{e^{i\phi}\cos\theta\sin\theta}{\sqrt{2}} & \cos^2\theta + \frac{\sin^2\theta}{2} - \frac{g\mu_B B}{D} \end{pmatrix}. \end{aligned} \tag{20}$$

In the rotating frame, the Hamiltonian of the NV center can be calculated by a unitary transformation,

$$\begin{aligned} H_{rot} &= U H_{lab} U^\dagger + i\partial_t U U^\dagger = e^{i\theta S_y} e^{i\phi S_z} H_{lab} e^{-i\phi S_z} e^{-i\theta S_y} + i\partial_t e^{i\theta S_y} e^{i\phi S_z} e^{-i\phi S_z} e^{-i\theta S_y} \\ &= \hbar \begin{pmatrix} D + g\mu_B B\cos\theta & -\frac{g\mu_B B\sin\theta}{\sqrt{2}} & 0 \\ -\frac{g\mu_B B\sin\theta}{\sqrt{2}} & 0 & -\frac{g\mu_B B\sin\theta}{\sqrt{2}} \\ 0 & -\frac{g\mu_B B\sin\theta}{\sqrt{2}} & D - g\mu_B B\cos\theta \end{pmatrix} + \hbar \begin{pmatrix} -\omega_r\cos\theta & \frac{\omega_r\sin\theta}{\sqrt{2}} & 0 \\ \frac{\omega_r\sin\theta}{\sqrt{2}} & 0 & \frac{\omega_r\sin\theta}{\sqrt{2}} \\ 0 & \frac{\omega_r\sin\theta}{\sqrt{2}} & \omega_r\cos\theta \end{pmatrix}, \end{aligned} \tag{21}$$

where the unitary operator is defined as $U = e^{i\theta S_y} e^{i\phi S_z}$. The second term on the right side of Eq. 21 represents Zeeman interaction arising from the pseudo-magnetic field due to the rotation of the NV center. In the case of an adiabatic process, $\omega_r \ll D - g\mu_B B\cos\theta$, the second term is significantly weaker than the first term, and can be treated as a perturbation. We neglect the off-diagonal terms in the first component since $g\mu_B B \ll D$, which are too small to induce significant mixing of the NV spin states.

$$H_{rot} \approx \hbar \begin{pmatrix} D + g\mu_B B\cos\theta & 0 & 0 \\ 0 & 0 & 0 \\ 0 & 0 & D - g\mu_B B\cos\theta \end{pmatrix} + \hbar \begin{pmatrix} -\omega_r\cos\theta & \frac{\omega_r\sin\theta}{\sqrt{2}} & 0 \\ \frac{\omega_r\sin\theta}{\sqrt{2}} & 0 & \frac{\omega_r\sin\theta}{\sqrt{2}} \\ 0 & \frac{\omega_r\sin\theta}{\sqrt{2}} & \omega_r\cos\theta \end{pmatrix}. \tag{22}$$

Therefore, the Hamiltonian in the rotating frame possesses three eigenstates, $|m_s\rangle_{rot}$ ($m_s = 0, \pm 1$):

$$|+1\rangle_{rot} = \begin{pmatrix} 1 \\ 0 \\ 0 \end{pmatrix}$$
$$|0\rangle_{rot} = \begin{pmatrix} 0 \\ 1 \\ 0 \end{pmatrix} \quad , \tag{23}$$
$$|-1\rangle_{rot} = \begin{pmatrix} 0 \\ 0 \\ 1 \end{pmatrix}$$

and the corresponding eigenvalues are $\hbar\left(D + g\mu_B B\cos\theta\right)$, 0, $\hbar\left(D + g\mu_B B\cos\theta\right)$, respectively. The new Hamiltonian in the laboratory frame can be transformed by applying the rotation transformation $R\left(t\right) = e^{-i\phi S_z}e^{-i\theta S_y}$,

$$\begin{aligned} H_{lab} &= RH_{rot}R^{\dagger} + i\partial_t RR^{\dagger} = e^{-i\phi S_z}e^{-i\theta S_y}H_{rot}e^{i\theta S_y}e^{i\phi S_z} + i\partial_t e^{-i\phi S_z}e^{-i\theta S_y}e^{i\theta S_y}e^{i\phi S_z} \\ &= \hbar\begin{pmatrix} D\frac{1+\cos^2\theta}{2} + g\mu_B B\cos^2\theta & \frac{e^{-i\phi}(D+g\mu_B B)\cos\theta\sin\theta}{\sqrt{2}} & \frac{e^{-2i\phi}D\sin^2\theta}{2} \\ \frac{e^{i\phi}(D+g\mu_B B)\cos\theta\sin\theta}{\sqrt{2}} & D\sin^2\theta & -\frac{e^{-i\phi}(D-g\mu_B B)\cos\theta\sin\theta}{\sqrt{2}} \\ \frac{e^{2i\phi}D\sin^2\theta}{2} & -\frac{e^{i\phi}(D-g\mu_B B)\cos\theta\sin\theta}{\sqrt{2}} & D\frac{1+\cos^2\theta}{2} - g\mu_B B\cos^2\theta \end{pmatrix} \end{aligned} \quad . \tag{24}$$

The eigenstates of the Hamiltonian in the laboratory frame also can be calculated through the rotation transformation,

$$|+1, t\rangle_{lab} = R\left(t\right)|+1\rangle_{rot} = \begin{pmatrix} e^{-i\phi}\cos^2\frac{\theta}{2} \\ \frac{\sin\theta}{\sqrt{2}} \\ e^{i\phi}\sin^2\frac{\theta}{2} \end{pmatrix}$$
$$|0, t\rangle_{lab} = R\left(t\right)|0\rangle_{rot} = \begin{pmatrix} -\frac{e^{-i\phi}\sin\theta}{\sqrt{2}} \\ \cos\theta \\ \frac{e^{i\phi}\sin\theta}{\sqrt{2}} \end{pmatrix} \quad , \tag{25}$$
$$|-1, t\rangle_{lab} = R\left(t\right)|-1\rangle_{rot} = \begin{pmatrix} e^{-i\phi}\sin^2\frac{\theta}{2} \\ -\frac{\sin\theta}{\sqrt{2}} \\ e^{i\phi}\cos^2\frac{\theta}{2} \end{pmatrix}$$

which are same as the eigenstates of the Hamiltonian without the external magnetic field (Eq. 12). Thus, the Berry phase of the rotating NV center is:

$$\gamma_{m_s} = m_s \omega_r t \cos\theta \tag{26}$$

.

Similarly, the interaction between microwave and the time-dependent spin states is still divided into two components. For the longitudinal component, it can be expressed as

$$\begin{aligned}
&{}_{lab}\langle \pm 1, t| e^{iH_{lab}t/\hbar} e^{-i\gamma_{\pm 1}} H_{MW,z,lab} e^{i\gamma_0} e^{-iH_{lab}t/\hbar} |0, t\rangle_{lab} \\
&= g\mu_B B_{MW} \cos(\omega_{MW} t) \cos\theta' {}_{lab}\langle \pm 1, 0| e^{i\theta S_y} e^{i\phi S_z} e^{iH_{lab}t/\hbar} e^{-i\gamma_{\pm 1}} S_z e^{i\gamma_0} e^{-iH_{lab}t/\hbar} e^{-i\phi S_z} e^{-i\theta S_y} |0, 0\rangle_{lab} \\
&= g\mu_B B_{MW} \cos(\omega_{MW} t) \cos\theta' e^{-i(\gamma_{\pm 1} - \gamma_0)} e^{i\left(E_{B,\pm 1} - E_{B,0}\right)t/\hbar} {}_{lab}\langle \pm 1, 0| e^{i\theta S_y} e^{i\phi S_z} S_z e^{-i\phi S_z} e^{-i\theta S_y} |0, 0\rangle_{lab} \\
&= \tfrac{1}{2} g\mu_B B_{MW} \cos\theta' \left(e^{i\omega_{MW} t} + e^{-i\omega_{MW} t}\right) e^{\mp i\omega_r t \cos\theta} e^{i(D \pm g\mu_B B \cos\theta)t} {}_{lab}\langle \pm 1, 0| e^{i\theta S_y} S_z e^{-i\theta S_y} |0, 0\rangle_{lab} \quad , \\
&= \tfrac{1}{2} g\mu_B B_{MW} \cos\theta' \left[e^{i(\omega_{MW} \mp \omega_r \cos\theta + D \pm g\mu_B B \cos\theta)t} + e^{i(-\omega_{MW} \mp \omega_r \cos\theta + D \pm g\mu_B B \cos\theta)t}\right] \\
&\times {}_{lab}\langle \pm 1, 0| e^{i\theta S_y} S_z e^{-i\theta S_y} |0, 0\rangle_{lab} \\
&\approx \tfrac{1}{2} g\mu_B B_{MW} \cos\theta' e^{i(-\omega_{MW} + D \pm g\mu_B B \cos\theta \mp \omega_r \cos\theta)t} {}_{lab}\langle \pm 1, 0| e^{i\theta S_y} S_z e^{-i\theta S_y} |0, 0\rangle_{lab}
\end{aligned} \tag{27}$$

where the E_{B,m_s} is the eigenvalue of the Hamiltonian H_{lab} for the spin state $|m_s, t\rangle_{lab}$. The spin resonance frequency, transformed from $|m_s = 0\rangle_{lab}$ to $|m_s = \pm 1\rangle_{lab}$ is $D \pm g\mu_B B \cos\theta \mp \omega_r \cos\theta$. The frequency shift due to the Berry phase is $\mp\omega_r \cos\theta$.

The transverse component can be expressed as

$$\begin{aligned}
&{}_{lab}\langle \pm 1, t| e^{iH_{lab}t/\hbar} e^{-i\gamma_{\pm 1}} H_{MW,y,lab} e^{i\gamma_0} e^{-iH_{lab}t/\hbar} |0, t\rangle_{lab} \\
&= g\mu_B B_{MW} \cos(\omega_{MW} t) \sin\theta' {}_{lab}\langle \pm 1, 0| e^{i\theta S_y} e^{i\phi S_z} e^{iH_{lab}t/\hbar} e^{-i\gamma_{\pm 1}} S_y e^{i\gamma_0} e^{-iH_{lab}t/\hbar} e^{-i\phi S_z} e^{-i\theta S_y} |0, 0\rangle_{lab} \\
&= g\mu_B B_{MW} \cos(\omega_{MW} t) \sin\theta' e^{-i(\gamma_{\pm 1} - \gamma_0)} e^{i\left(E_{B,\pm 1} - E_{B,0}\right)t/\hbar} {}_{lab}\langle \pm 1, 0| e^{i\theta S_y} e^{i\phi S_z} S_y e^{-i\phi S_z} e^{-i\theta S_y} |0, 0\rangle_{lab} \, . \\
&= \tfrac{1}{2} g\mu_B B_{MW} \sin\theta' \left(e^{i\omega_{MW} t} + e^{-i\omega_{MW} t}\right) e^{\mp i\omega_r t \cos\theta} e^{i(D \pm g\mu_B B \cos\theta)t} \\
&\times {}_{lab}\langle \pm 1, 0| e^{i\theta S_y} \tfrac{1}{2i} \left(S_+ e^{i\omega_r t} - S_- e^{-i\omega_r t}\right) e^{-i\theta S_y} |0, 0\rangle_{lab}
\end{aligned} \tag{28}$$

The expected values are written as

$$\begin{aligned}
&{}_{lab}\langle +1, t| e^{iH_{lab}t/\hbar} e^{-i\gamma_{+1}} H_{MW,y,lab} e^{i\gamma_0} e^{-iH_{lab}t/\hbar} |0, t\rangle_{lab} \\
&= \tfrac{1}{4i} g\mu_B B_{MW} \sin\theta' \left[e^{i(\omega_{MW} - \omega_r \cos\theta + D + g\mu_B B \cos\theta + \omega_r)t} + e^{i(-\omega_{MW} - \omega_r \cos\theta + D + g\mu_B B \cos\theta + \omega_r)t}\right] \quad , \\
&\times {}_{lab}\langle +1, 0| e^{i\theta S_y} S_+ e^{-i\theta S_y} |0, 0\rangle_{lab} \\
&\approx \tfrac{1}{4i} g\mu_B B_{MW} \sin\theta' e^{i(-\omega_{MW} - \omega_r \cos\theta + D + g\mu_B B \cos\theta + \omega_r)t} {}_{lab}\langle +1, 0| e^{i\theta S_y} S_+ e^{-i\theta S_y} |0, 0\rangle_{lab}
\end{aligned} \tag{29}$$

$$
\begin{aligned}
&{}_{lab}\langle -1,t|\, e^{iH_{lab}t/\hbar} e^{-i\gamma_{-1}} H_{MW,y,lab} e^{i\gamma_0} e^{-iH_{lab}t/\hbar} |0,t\rangle_{lab} \\
&= -\tfrac{1}{4i} g\mu_B B_{MW} \sin\theta' \left[e^{i(\omega_{MW}+\omega_r\cos\theta+D-g\mu_B B\cos\theta-\omega_r)t} + e^{i(-\omega_{MW}+\omega_r\cos\theta+D-g\mu_B B\cos\theta-\omega_r)t} \right] \\
&\times {}_{lab}\langle -1,0|\, e^{i\theta S_y} S_- e^{-i\theta S_y} |0,0\rangle_{lab} \\
&\approx -\tfrac{1}{4i} g\mu_B B_{MW} \sin\theta' e^{i(-\omega_{MW}+\omega_r\cos\theta+D-g\mu_B B\cos\theta-\omega_r)t} {}_{lab}\langle -1,0|\, e^{i\theta S_y} S_- e^{-i\theta S_y} |0,0\rangle_{lab}
\end{aligned}
\quad (30)
$$

The transformation resonance frequency is $D \pm g\mu_B B\cos\theta \pm \omega_r(1-\cos\theta)$ between the $|m_s=0\rangle_{lab}$ state and $|m_s=\pm1\rangle_{lab}$ state. The corresponding frequency shift duo to the Berry phase also is $\pm\omega_r\cos\theta$, and the frequency shift induced by the rotational Doppler effect is ω_r. Similar to the case of zero external magnetic field, the predominant transition probability arises from the longitudinal component, characterized by a frequency shift of $\mp\omega_r\cos\theta$ due to the Berry phase.

Supplementary Figure 6(c) shows the frequency shift induced by the Berry phase in a levitated nanodiamond rotating counterclockwisely (viewed from the positive z direction unless otherwise specified). The external magnetic field along the z direction is about 100 G. The resonance frequency transition between $|m_s=0\rangle_{lab}$ state and $|m_s=+1\rangle_{lab}$ state decreases with an increasing of the rotation frequency, in contrast to the behavior observed in the levitated nanodiamond rotating clockwise. The red curve is the theoretical calculation for the angle of $\theta = 21.5°$ between the NV axis and the rotating axis. The experimental data is in good agreement with the theoretical calculation, suggesting a consistent orientation of the NV centers at various rotation frequencies.

C. Pseudo-magnetic field due to rotation

The Berry phase observed in the laboratory frame is equivalent to the pseudo-magnetic field (called the Barnett field in [8]) in the rotational frame. In our experiment, the mircowave source is fixed in the laboratory frame. Only the levitated nanodiamond is rotating. Thus, we observe the effect of the Berry phase [8]. It will be beneficial to also consider this system in the rotational frame. The electron spin resonance frequency shift of the rotating NV center involves the combination of the pseudo-magnetic field and the rotational Doppler effect in the rotating frame. As expressed in Eq. 21, the Hamiltonian of the pseudo-magnetic field in

the rotating frame, induced by the rotation of a diamond particle, can be given by

$$H_{\omega_r} = \hbar \begin{pmatrix} -\omega_r \cos\theta & \frac{\omega_r \sin\theta}{\sqrt{2}} & 0 \\ \frac{\omega_r \sin\theta}{\sqrt{2}} & 0 & \frac{\omega_r \sin\theta}{\sqrt{2}} \\ 0 & \frac{\omega_r \sin\theta}{\sqrt{2}} & \omega_r \cos\theta \end{pmatrix} \approx \hbar \begin{pmatrix} -\omega_r \cos\theta & 0 & 0 \\ 0 & 0 & 0 \\ 0 & 0 & \omega_r \cos\theta \end{pmatrix}, \tag{31}$$

where the off-diagonal terms also can be ignored. So the Hamiltonian of the NV center in the rotating frame can be expressed as

$$H_{rot} = \hbar \begin{pmatrix} D + g\mu_B B\cos\theta - \omega_r \cos\theta & 0 & 0 \\ 0 & 0 & 0 \\ 0 & 0 & D - g\mu_B B\cos\theta + \omega_r \cos\theta \end{pmatrix}. \tag{32}$$

The corresponding eigenvalues are $\hbar\left(D + g\mu_B B\cos\theta - \omega_r\cos\theta\right)$, 0, $\hbar\left(D - g\mu_B B\cos\theta + \omega_r\cos\theta\right)$ for spin state $|+1\rangle_{rot}$, $|0\rangle_{rot}$ and $|-1\rangle_{rot}$, respectively.

The Hamiltonian describing the interaction of the microwave term with the NV center in the rotating frame can be expressed as

$$\begin{aligned} &H_{MW,rot} = UH_{MW,lab}U^{\dagger} = e^{i\theta S_y} e^{i\phi S_z} H_{MW,lab} e^{-i\phi S_z} e^{-i\theta S_y} \\ &= g\mu_B B_{MW} \cos\left(\omega_{MW} t\right) e^{i\theta S_y} e^{i\phi S_z} \left(S_z \cos\theta' + S_y \sin\theta'\right) e^{-i\phi S_z} e^{-i\theta S_y} = H_{MW,z,rot} + H_{MW,y,rot} \end{aligned}, \tag{33}$$

where the longitudinal component is $H_{MW,z,rot} = g\mu_B B_{MW}\cos\left(\omega_{MW}t\right) e^{i\theta S_y} e^{i\phi S_z} S_z \cos\theta' e^{-i\phi S_z} e^{-i\theta S_y}$, and the transverse component is $H_{MW,y,rot} = g\mu_B B_{MW}\cos\left(\omega_{MW}t\right) e^{i\theta S_y} e^{i\phi S_z} S_y \sin\theta' e^{-i\phi S_z} e^{-i\theta S_y}$. The expected value of the spin state, interacting with the longitudinal component of microwave, can be expressed as

$$\begin{aligned} &{}_{rot}\langle \pm 1| e^{iH_{rot}t/\hbar} H_{MW,z,rot} e^{-iH_{rot}t/\hbar} |0\rangle_{rot} \\ &= g\mu_B B_{MW} \cos\theta' \cos\left(\omega_{MW}t\right)_{rot} \langle \pm 1| e^{iH_{rot}t/\hbar} e^{i\theta S_y} e^{i\phi S_z} S_z e^{-i\phi S_z} e^{-i\theta S_y} e^{-iH_{rot}t/\hbar} |0\rangle_{rot} \\ &= g\mu_B B_{MW} \cos\theta' \cos\left(\omega_{MW}t\right) e^{i\left(E_{\pm1,rot} - E_{0,rot}\right)t/\hbar}{}_{rot} \langle \pm 1| e^{i\theta S_y} e^{i\phi S_z} S_z e^{-i\phi S_z} e^{-i\theta S_y} |0\rangle_{rot} \\ &= \tfrac{1}{2} g\mu_B B_{MW} \cos\theta' \left(e^{i\omega_{MW}t} + e^{-i\omega_{MW}t}\right) e^{i\left(D \pm g\mu_B B\cos\theta \mp \omega_r \cos\theta\right)t}{}_{rot} \langle \pm 1| e^{i\theta S_y} S_z e^{-i\theta S_y} |0\rangle_{rot}, \\ &= \tfrac{1}{2} g\mu_B B_{MW} \cos\theta' \left[e^{i\left(\omega_{MW} + D \pm g\mu_B B\cos\theta \mp \omega_r\cos\theta\right)t} + e^{i\left(-\omega_{MW} D \pm g\mu_B B\cos\theta \mp \omega_r \cos\theta\right)t}\right] \\ &\times{}_{rot} \langle \pm 1| e^{i\theta S_y} S_z e^{-i\theta S_y} |0\rangle_{rot} \\ &\approx \tfrac{1}{2} g\mu_B B_{MW} \cos\theta' e^{i\left(-\omega_{MW} D \pm g\mu_B B\cos\theta \mp \omega_r\cos\theta\right)t}{}_{rot} \langle \pm 1| e^{i\theta S_y} S_z e^{-i\theta S_y} |0\rangle_{rot} \end{aligned} \tag{34}$$

where the $E_{m_s,rot}$ is the eigenvalue of the Hamiltonian H_{rot} for the spin state $|m_s\rangle_{rot}$ in the rotating frame. The transformation resonance frequency is $D \pm g\mu_B B\cos\theta \mp \omega_r\cos\theta$,

and the last term $\mp\omega_r \cos\theta$ is the frequency shift due to the pseudo-magnetic field. The transverse component can be expressed as

$$\begin{aligned}
&{}_{rot}\langle\pm1|\, e^{iH_{rot}t/\hbar} H_{MW,y,rot} e^{-iH_{rot}t/\hbar} |0\rangle_{rot} \\
&= g\mu_B B_{MW} \sin\theta' \cos(\omega_{MW}t)\, {}_{rot}\langle\pm1|\, e^{iH_{rot}t/\hbar} e^{i\theta S_y} e^{i\phi S_z} S_y e^{-i\phi S_z} e^{-i\theta S_y} e^{-iH_{rot}t/\hbar} |0\rangle_{rot} \\
&= g\mu_B B_{MW} \sin\theta' \cos(\omega_{MW}t)\, e^{i(E_{\pm1,rot}-E_{0,rot})t/\hbar}\, {}_{rot}\langle\pm1|\, e^{i\theta S_y} e^{i\phi S_z} S_y e^{-i\phi S_z} e^{-i\theta S_y} |0\rangle_{rot} . \\
&= \tfrac{1}{2} g\mu_B B_{MW} \sin\theta' \left(e^{i\omega_{MW}t} + e^{-i\omega_{MW}t}\right) e^{i(D\pm g\mu_B B\cos\theta \mp\omega_r\cos\theta)t} \\
&\times {}_{rot}\langle\pm1|\, e^{i\theta S_y} \tfrac{1}{2i}\left(S_+ e^{i\omega_r t} - S_- e^{-i\omega_r t}\right) e^{-i\theta S_y} |0\rangle_{rot}
\end{aligned} \tag{35}$$

The expected values are written as

$$\begin{aligned}
&{}_{rot}\langle+1|\, e^{iH_{rot}t/\hbar} H_{MW,y,rot} e^{-iH_{rot}t/\hbar} |0\rangle_{rot} \\
&= \tfrac{1}{4i} g\mu_B B_{MW} \sin\theta' \left[e^{i(\omega_{MW}+D+g\mu_B B\cos\theta-\omega_r\cos\theta+\omega_r)t} + e^{i(-\omega_{MW}+D+g\mu_B B\cos\theta-\omega_r\cos\theta+\omega_r)t}\right] \\
&\times {}_{rot}\langle+1|\, e^{i\theta S_y} S_+ e^{-i\theta S_y} |0\rangle_{rot} \\
&\approx \tfrac{1}{4i} g\mu_B B_{MW} \sin\theta' e^{i(-\omega_{MW}+D+g\mu_B B\cos\theta-\omega_r\cos\theta+\omega_r)t}\, {}_{rot}\langle+1|\, e^{i\theta S_y} S_+ e^{-i\theta S_y} |0\rangle_{rot}
\end{aligned} , \tag{36}$$

$$\begin{aligned}
&{}_{rot}\langle-1|\, e^{iH_{rot}t/\hbar} H_{MW,y,rot} e^{-iH_{rot}t/\hbar} |0\rangle_{rot} \\
&= -\tfrac{1}{4i} g\mu_B B_{MW} \sin\theta' \left[e^{i(\omega_{MW}+D-g\mu_B B\cos\theta+\omega_r\cos\theta-\omega_r)t} + e^{i(-\omega_{MW}+D-g\mu_B B\cos\theta+\omega_r\cos\theta-\omega_r)t}\right] \\
&\times {}_{rot}\langle-1|\, e^{i\theta S_y} S_- e^{-i\theta S_y} |0\rangle_{rot} \\
&\approx -\tfrac{1}{4i} g\mu_B B_{MW} \sin\theta' e^{i(-\omega_{MW}+D-g\mu_B B\cos\theta+\omega_r\cos\theta-\omega_r)t}\, {}_{rot}\langle+1|\, e^{i\theta S_y} S_+ e^{-i\theta S_y} |0\rangle_{rot}
\end{aligned} . \tag{37}$$

The transformation resonance frequency between the $|m_s = 0\rangle_{rot}$ state and $|m_s = \pm1\rangle_{rot}$ state is $D \pm g\mu_B B\cos\theta \pm \omega_r(1-\cos\theta)$. The frequency shift of $\pm\omega_r(1-\cos\theta)$ is induced by both the pseudo-magnetic field and the rotational Doppler effect [8] in the rotating frame.

SUPPLEMENTARY NOTE 5: QUANTUM MEASUREMENT OF LEVITATED DIAMOND NV CENTERS

The power of 532 nm laser for NV initialization is very weak to minimize laser heating in high vacuum, leading to a long NV polarization time. The measured initialization time, shown in Supplementary Figure 7(a), is 1.05 ms at the 532 nm laser intensity of 0.113 W/mm^2. We measure the spin relaxation time (T_1) of the levitated nanodiamond in Supplementary Figure 7(c), indicating $T_1 = 3.60$ ms. It is three times longer than the initialization time. The T_2^* and T_2 are measured, as shown in Supplementary Figure 7(c) and (d). The

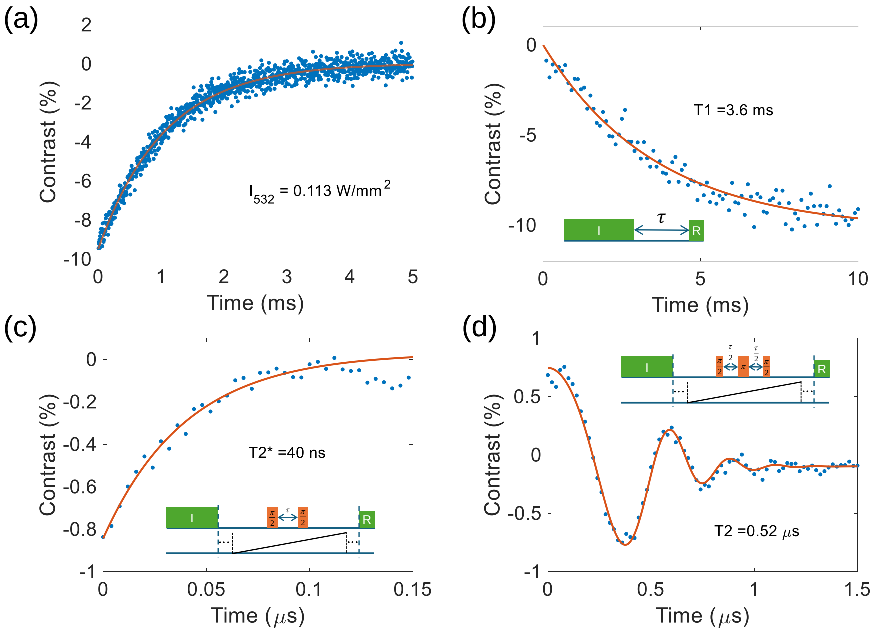

Supplementary Figure 7. Quantum measurement of NV centers in a levitated nanodiamond. (a) Initialization time of levitated Nv centers when the 532 nm laser intensity is 0.113 W/mm^2. The initialization time is 1.05 ms. (b) Experimental result of T_1 measurement of the levitated nanodiamond. The insert is the sequence of T_1 measurement. The T_1 is 3.6 ms. (c) Ramsey measurement and its corresponding sequence with a T_2^* of 40 ns. (d) Spin Echo measurement and its corresponding sequence with a T_2 of 0.52 μs. The oscillation is induced by the misalignment of the magnetic field with the rotation axis. In the sequences, the green and orange regions represent the pulses of the 532 nm laser and the microwave, respectively. The black lines are the rotation phase.

T_2^* is 40 ns and the T_2 is 0.52 μs. In the spin echo measurement, the oscillation is a result of the misalignment of the magnetic field with the rotation axis [9].

Due to the Ω-shape design of the microwave antenna, the orientation of the magnetic component of microwave is located in yz-plane and slightly away from the z axis with an angle about $\theta' = 8.5°$. Thus, the effective magnetic field $B_{MW}^{\perp}$ of the microwave acting on NV spins keeps varying at different rotation phase $\phi(t)$ of the levitated nanodiamond. We set the direction of a NV spin to be $\mathbf{n}_{NV} = (\sin\theta, 0, \cos\theta)$ in the xz-plane at initial time

$(t = 0)$, and then rotate it around the z axis. The rotation matrix is:

$$\mathbf{r}_z = \begin{pmatrix} \cos\phi(t) & -\sin\phi(t) & 0 \\ \sin\phi(t) & \cos\phi(t) & 0 \\ 0 & 0 & 1 \end{pmatrix}. \tag{38}$$

After a rotation time of t, the direction of the NV spin is changed to

$$\mathbf{n}'_{NV} = (\cos\phi(t)\sin\theta, \sin\phi(t)\sin\theta, \cos\theta). \tag{39}$$

The unit vector of the direction of the magnetic field of microwave is $\mathbf{n}_{MW} = (0, -\sin\theta', \cos\theta')$. So the angle between the NV spin and the microwave is $\arccos(\cos\theta\cos\theta' - \sin\phi(t)\sin\theta\sin\theta')$. The effective magnetic field of microwave is

$$B_{MW}^{\perp} = B_{MW}\sqrt{1 - (\cos\theta\cos\theta' - \sin\phi(t)\sin\theta\sin\theta')^2}. \tag{40}$$

Rabi frequency is proportional to the effective magnetic field of microwave, $\Omega_{Rabi} \propto B_{MW}^{\perp}$. Therefore, it is necessary to synchronize the measurement cycle and the rotation signal of the levitated nanodiamond, and apply microwave pulse at the same rotation phase in repeated Rabi oscillation measurements.

SUPPLEMENTARY NOTE 6: NANODIAMOND FIXED ON GLASS

To compare with a levitated nanodiamond, we carry out the quantum measurement of a nanodiamond fixed on a glass cover slip, with a thickness of 300 μm. The glass cover slip is placed at the center of the surface ion trap to keep the direction and power of microwave unchanged compared to a levitated nanodiamond. Supplementary Figure 8(a) is the ODMR of the nanodiamond. The linewidth is smaller than that of a levitated nanodiamond. The initialization time are measured at $I_{532} = 0.126$ W/mm^2 (blue circles) and $I_{532} = 31.8$ W/mm^2 (red squares in Supplementary Figure 8(b)) for comparison. The initialization times are 0.469 ms and 1.77 μs, respectively. T1 of the nanodiamond on glass surface is 2.34 ms (Supplementary Figure 8(c)), which is close to that of a levitated one. We measure Rabi oscillation at weak 532 nm laser ($I_{532} = 0.126$ W/mm^2), which is similar to the intensity used for a levitated nanodiamond in high vacuum (Supplementary Figure 8(d)). We get the similar result with high intensity of 532 nm laser ($I_{532} = 31.8$ W/mm^2). The Rabi frequency is 1.99 MHz and the decay time T_2^{Rabi} are 0.845 μs and 0.904 μs, respectively.

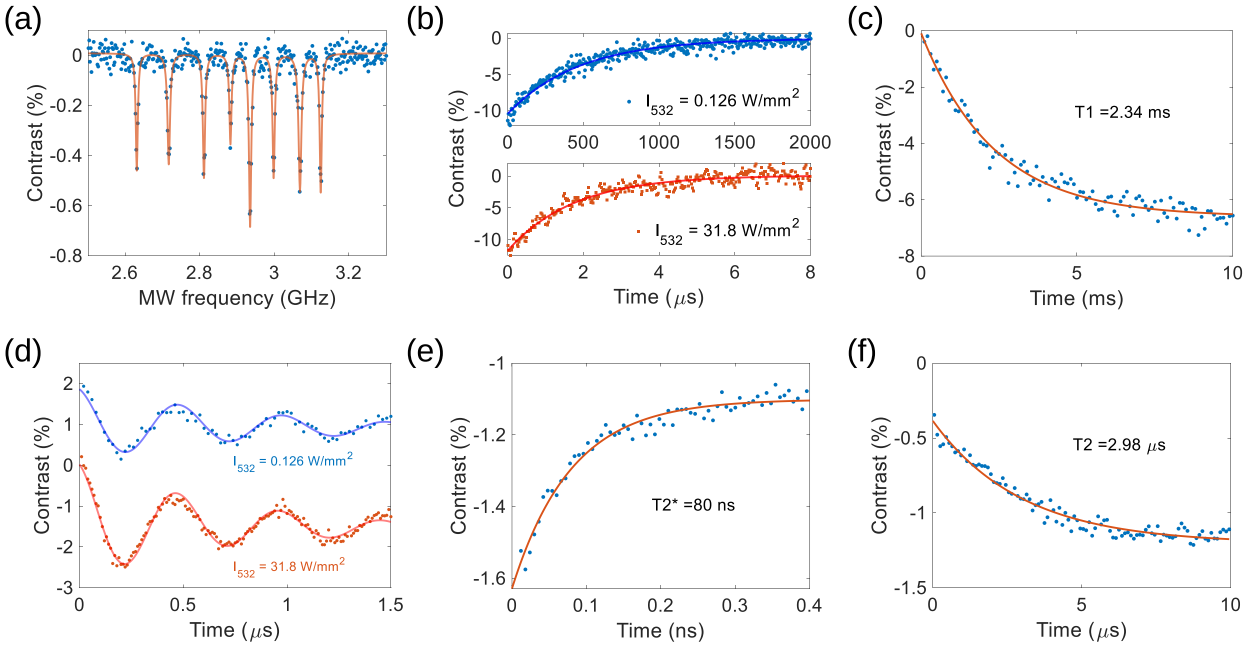

Supplementary Figure 8. Quantum measurement of NV centers in a nanodiamond fixed on a glass. (a) ODMR of the fixed NV centers. (b) Initialization of the nanodiamond Nv centers when the 532 nm laser intensity is 0.126 W/mm^2 (blue circles) or 31.8 W/mm^2 (red squares). The initialization times are 0.469 ms and 1.77 μs, respectively. (c) Experimental T_1 measurement of the nanodiamond. The T_1 is 2.34 ms. (d) Rabi oscillations of the nanodiamond when the 532 nm laser intensity is 0.126 W/mm^2 (blue circles) or 31.8 W/mm^2 (red squares). The Rabi frequencies are both 1.99 MHz and the decay times $T_2^{\rm rabi}$ are 0.845 μs and 0.904 μs, respectively. The blue curve is shifted 2% to separate the curves. (e) Experimental Ramsey measurement. The T_2^* of the nanodiamond NV centers fixed on a glass is 80 ns. (f) Spin Echo measurement, which indicates a T_2 of 2.98 μs.

Supplementary Figure 8(e) shows the Ramsey measurement, while Supplementary Figure 8(f) is the spin echo measurement. The corresponding values for T_2^* and T_2 are 80 ns and 2.98 μs, respectively.

[1] T. H. Kim, P. F. Herskind, T. Kim, J. Kim, and I. L. Chuang, Surface-electrode point paul trap, Phys. Rev. A **82**, 043412 (2010).

[2] H.-K. Li, E. Urban, C. Noel, A. Chuang, Y. Xia, A. Ransford, B. Hemmerling, Y. Wang, T. Li, H. Häffner, and X. Zhang, Realization of translational symmetry in trapped cold ion rings, Phys. Rev. Lett. **118**, 053001 (2017).

[3] F. Liu, K. Daun, D. Snelling, and G. Smallwood, Heat conduction from a spherical nanoparticle: status of modeling heat conduction in laser-induced incandescence, Applied Physics B **83**, 355 (2006).

[4] D. E. Chang, C. A. Regal, S. B. Papp, D. J. Wilson, J. Ye, O. Painter, H. J. Kimble, and P. Zoller, Cavity opto-mechanics using an optically levitated nanosphere, Proceedings of the National Academy of Sciences **107**, 1005 (2010).

[5] Y. Jin, J. Yan, S. J. Rahman, J. Li, X. Yu, and J. Zhang, 6 ghz hyperfast rotation of an optically levitated nanoparticle in vacuum, Photon. Res. **9**, 1344 (2021).

[6] J. Fremerey, Spinning rotor vacuum gauges, Vacuum **32**, 685 (1982).

[7] D. Maclaurin, M. W. Doherty, L. C. L. Hollenberg, and A. M. Martin, Measurable quantum geometric phase from a rotating single spin, Phys. Rev. Lett. **108**, 240403 (2012).

[8] H. Chudo, M. Matsuo, S. Maekawa, and E. Saitoh, Barnett field, rotational doppler effect, and berry phase studied by nuclear quadrupole resonance with rotation, Phys. Rev. B **103**, 174308 (2021).

[9] J. R. Maze, P. L. Stanwix, J. S. Hodges, S. Hong, J. M. Taylor, P. Cappellaro, L. Jiang, M. V. G. Dutt, E. Togan, A. S. Zibrov, A. Yacoby, R. L. Walsworth, and M. D. Lukin, Nanoscale magnetic sensing with an individual electronic spin in diamond, Nature **455**, 644 (2008).
